# Molecular Epidemiology of Japanese Encephalitis Virus, Taiwan

**DOI:** 10.3201/eid1605.091055

**Published:** 2010-05

**Authors:** Jyh-Hsiung Huang, Ting-Hsiang Lin, Hwa-Jen Teng, Chien-Ling Su, Kun-Hsien Tsai, Liang-Chen Lu, Cheo Lin, Cheng-Fen Yang, Shu-Fen Chang, Tsai-Ling Liao, Sheng-Kai Yu, Chia-Hsin Cheng, Mei-Chun Chang, Huai-Chin Hu, Pei-Yun Shu

**Affiliations:** Centers for Disease Control, Taipei, Taiwan (J.-H. Huang, T.-H. Lin, H.-J. Teng, C.-L. Su, L.-C. Lu, C. Lin, C.-F. Yang, S.-F. Chang, T.-L. Liao, S.-K. Yu, C.-H. Cheng, M.-C. Chang, H.-C. Hu, P.-Y. Shu); National Taiwan University, Taipei (K.-H. Tsai)

**Keywords:** Japanese encephalitis virus, epidemiology, viruses, genotype, Taiwan, letter

**To the Editor:** Japanese encephalitis virus (JEV) is a mosquito-borne member of the family *Flaviviridae* and the genus *Flavivirus*. JEV is a major cause of viral encephalitis in Asia. Phylogenetic analysis of the envelope (E) gene sequences has shown that JEV strains can be clustered into 5 distinct genotypes (*1*). Among them, genotype III (GIII) has had the widest geographic distribution in countries in Asia, including Japan, South Korea, People’s Republic of China, Taiwan, Vietnam, the Philippines, and India (*2*). Before 1990, GIII had been the major epidemic JEV type in these areas. However, the introduction of JEV genotype I (GI) has been reported in Japan, Vietnam, South Korea, Thailand, and China in the past decade (*3*–*6*). Nabeshima et al. recently reported surveillance results that provided substantial evidence of frequent introductions of JEV GI into Japan from Southeast Asia and continental eastern Asia (*7*). Because all current vaccines are derived from JEV GIII strains, the effectiveness of vaccination in inducing protective neutralizing antibodies against various genotype strains needs to be carefully evaluated, taking into account genotype shift in these countries.

Japanese encephalitis is endemic in Taiwan. Reports on the molecular epidemiology of JEV in Taiwan are scarce. Jan et al. (*8*) reported the genetic variation of 47 JEV isolates from Taiwan before 1994. Phylogenetic analysis showed that all Taiwanese isolates were GIII, and they were classified into 3 clusters.

To understand the genetic variation of JEV strains currently circulating in Taiwan, we conducted a surveillance program in the following areas: northern (Taipei, Taoyuan, and Yilan counties and Taipei City), central (Taichung and Changhua counties), southern (Tainan and Kaohsiung counties), and eastern (Hualien County) during 2005–2008. Real-time reverse transcription–PCR (RT-PCR) was used to screen JEV in mosquito pools, pig serum specimens, and human cerebrospinal fluid as described (*9*). Mosquitoes were pooled by species, location, and collection date in groups of 30–50 mosquitoes. Mosquito pools were homogenized and clarified by centrifugation, and the supernatants were sterilized by filtration and removed for real-time RT-PCR and virus isolation.

We used 3 sets of primers for real-time RT-PCR: flavivirus-specific (FL-F1: 5′-GCCATATGG TACATGTGGCTGGGAGC-3′; FL-R3: 5′-GTKATTCTTGTGTCCCAWCCGGCTGTGTCATC-3′; FL-R4: 5′-GTGATGCGRGTGTCCCAGCCRGCKGTGTCATC-3′), JEV-specific (*10*) (JE3F1: 5′-CCCTCAGAACCGTCTCGGAA-3′ and JE3R1: 5′-CTATTCCCAGGTGTCAATATGCTGT-3′), and JEV GIII–specific (E12F: 5′-CTGGGAATGGGCAATCGTG-3′ and E325R: 5′-TGTCAATGCTTCCCTTCCC-3′). Samples with positive results by RT-PCR were subjected to virus isolation by using a mosquito C6/36 cell line. A total of 47 JEV isolates were obtained: 38 from mosquitoes, 8 from pig serum samples, and 1 from human cerebrospinal fluid.

Viral RNA was extracted from JEV-infected culture medium, and RT-PCR and DNA sequencing were performed to determine the complete E gene sequences of JEV isolates. Multiple sequence alignment and phylogenetic analysis were conducted by using CLUSTALW software (www.ebi.ac.uk/Tools/clustalw2/index.html) and MEGA version 4 (www.megasofteware.net). The phylogenetic tree was constructed by the neighbor-joining method and the maximum composite likelihood model.

The Figure shows the phylogenetic tree derived from 67 samples of E gene sequences, including 28 representative new sequences in this study (GenBank accession nos. GQ260608–GQ260635), 10 sequences of Taiwanese strains isolated before 2002, and 29 sequences from GenBank. The results show that isolates from Taiwan comprised 2 genotypes, GIII and GI. All of the JEV isolates from Taiwan obtained during 2005–2008, except 2 strains (TPC0806c/M/2008 and YL0806f/M/2008), belonged to GIII and formed into 2 clusters.

**Figure:**
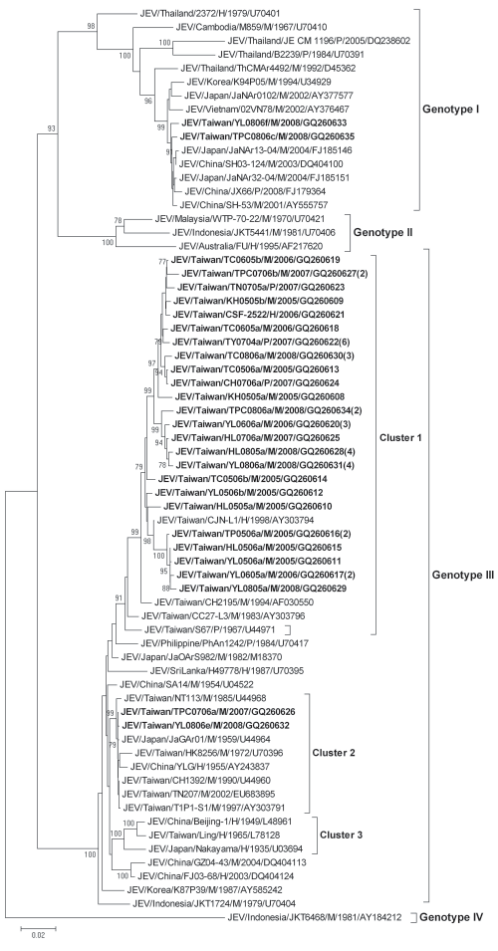
Phylogenetic tree showing the genetic relationship among Japanese encephalitis virus (JEV) isolates. The tree was constructed on the basis of complete envelope (E) nucleotide sequences of JEV strains. Sequences obtained in this study are indicated in **boldface**. Genotypes are indicated on the right. Viruses were identified by using the nomenclature of virus/country/strain/source/year of isolation/GenBank accession number. Numbers in parentheses indicate the number of isolates that showed 100% nucleotide homology. Isolates with the same sequences were collected at the same time from the same location in this study. Analysis was performed by using MEGA 4 software (www.megasoftware.net) and neighbor-joining (maximum composite likelihood) methods. Bootstrap support values >75 are shown (1,000 replicates). CH, Changhua County; HL, Hualien County; KH, Kaohsiung County; TC, Taichung County; TN, Tainan County; TP, Taipei County; TPC, Taipei City**;** TY, Taoyuan County; YL, Yilan County; M, mosquito pool; p, pig serum; H, human sample. Scale bar indicates nucleotide substitutions per site.

Cluster 1 contains most new isolates prevalent in different areas of Taiwan. Although cluster 1 isolates are closely related to other JEV strains from Asia, these isolates, together with previously published JEV sequences from Taiwan, form a distinct lineage and may have been continuously evolving and locally adapting in Taiwan. Cluster 2 contains only 2 new isolates, TPC0706a/M/2007 and YL0806e/M/2008, which were isolated from the *Culex tritaeniorhynchus* mosquito pools in Kuantu Nature Park, Taipei City, and from a pig farm in Wujie Township, Yilan County, respectively.

Notably, the 2 GI strains, TPC0806c/M/2008 and YL0806f/M/2008, were isolated from the same areas as the GIII cluster 2 strains. These areas are adjacent to the wetlands, which are stopover sites for migratory birds. These 2 GI strains are most closely related to the strains of the subcluster II JEV strains reported by Nabeshima et al. (*7*). The TPC0806c/M/2008 GI strain is most closely related to Japan/JaNAr13–04/M/2004 and China/SH03–124/M/2003 strains (99.5% and 99.4% identities, respectively), and the YL0806f/M/2008 GI strain is most closely related to Japan/JaNAr13–04/M/2004 and China/JX66/P/2008 strains (99.3% and 99.3% identities, respectively). Therefore, JEV GI strains from Taiwan were likely introduced by water birds migrating back and forth along the Asia–Australasia flyway, which passes through many countries, including Indonesia, Malaysia, Australia, the Philippines, Taiwan, China, and Japan (*3*).

Our results clearly showed that JEV GIII strains remain the most dominant population circulating in Taiwan, although 2 JEV GI strains were isolated from wetland areas in northern Taiwan in 2008. Further studies are needed to continuously monitor the changing epidemiologic pattern of JEV strains endemic in Taiwan and newly introduced viruses.
